# Dental Education in the Public Schools

**Published:** 1902-06-15

**Authors:** W. L. Mercer


### DENTAL EDUCATION IN THE PUBLIC SCHOOLS.

BY PROF. W. L. MERCER.

Read before the Southwestern Michigan Dental Society, April 9, 1902.

In beginning this paper the question that first comes to me is: What need is there of any special dental education in our schools that is not already provided for, and is this question of enough importance that we should use the time of this convention in its discussion?

It is quite generally agreed that our public schools have a most important function to perform in extending knowledge of the laws of public health, and, if modern society can support the fraternity of dental surgery, surely we should give it some attention in the school-room.

I will offer two principles which I believe will suggest a field of common interest to the pedagogue and the dentist and which I shall use to determine a line of thought for this paper.

1st. The public schools must meet the present general demand that they should furnish such knowledge and training to their pupils as will serve them best in the practical affairs of every day life. Wherever the man or woman may be called in later life to serve, in the State, in business, in society or in the home, he should be able to find some elements of his former schooling that shall enable him to meet with greater success these various responsibilities.

2d. Every professionally trained man or woman owes to the world and especially to his own little circle of influence such instructions and such example as his own particular advantages enable him to give.

By the co-operation of the dentists and teachers in the various cities and villages, some special instruction might be given to the pupils which would be for our mutual benefit and for the public good. The prime object in such instruction should be to teach the children to early form the habit of properly caring for their teeth and to awaken such an interest in their duty that the habit would become fixed and result in some degree in preserving and maintaining their health and beauty.

I need not discuss with you the importance and necessity of forming such a habit. It is a mark of good breeding. It is the sign of culture. It is an element in education. Our modern life makes it an absolute necessity. Much less ought I to discuss with you the necessity for instructing people as to the value of forming the habit of caring for the teeth.

The dental fraternity is supported in luxury because there is a lack of such care. The ignorance and careless neglect of parents is everywhere evident, although teachers and text-books on physiology and hygiene have emphasized the importance of properly caring for our bodies, yet there is still a need and growing demand for more of such instruction, especially in specific directions.

As far as special dental education in our common schools is concerned, it has been very informal and limited if not neglected altogether, and there is certainly a great opportunity for dental aid in such education.

The plan I shall outline is very simple, and possibly may already have been tried in a modified form at least.

By the co-operation of the local dentist and the superintendent of schools, arrangements can be made for a course of lectures or talks to be given to the pupils by the dentist. Our course of study is already much crowded and you will find that in most schools no elaborate plans can be carried out successfully. If the dentist could meet the pupils in the primary grades about four times a year, those of the grammar grades two or three times a year and the members of the high school once each year, a great deal of good could be accomplished and the regular work of the school would in no way be interfered with. He should bring with him such charts and specimens as he may have, and which might serve to awaken a deeper interest in the lessons he wishes to impress. The instructions given to the pupils at these times should be technical only in so far as it will further the end in view. At all of these exercises conducted by the dentist, the pupils should be listeners and questioners only. The teacher should be the note taker and gather from the lecture such facts as she may wish to use in reviews that should follow. Much will be suggested that can be used in the regular science and language work of the school and may be made a help to the other lessons.

I have suggested that more lessons be given to the primary grades than to the higher grades. Childhood is the great habit-forming period of our lives. It is then that impressions properly made on the mind have the greatest influence and may become a part of the character and nature of the person. Proper care for our bodies can be brought about only by correct habits of daily life. If these habits are formed in childhood you may be reasonably sure that the man will be able to preserve and maintain the health and vigor of his physical organism.

Why should the dentist give such instruction? His technical knowledge is much greater than that of the teacher. He is a stranger in the school-room and his reputation as being "the dentist," will serve to awaken the interest of the children from the first, and if he is able to gain their good will he can have the greatest influence over them. If you have never stepped into the school-room and talked to the children and questioned them in regard to their own particular way of caring for their teeth, you can not understand the influence for good you may have over them. You will find after one of your lectures that they will not only be particularly careful about the care of their teeth but will search for mouths with ill-kept teeth and will carry your lessons to people you will not be able to reach.

Beside the benefit that would come to the public in general by the course of instruction as outlined there is also the benefit to the dental profession. I am not acquainted with the history of dentistry but it must be quite a modern institution. Like the doctors of medicine you are a necessity because of the ills that human flesh is heir to. But because you prosper because of our aches, the burden of your work should be to teach people to avoid as far as possible the necess ty for your operations. And I know that you do. There is no class of men whose hearts can be made more glad by seeing a perfect set of teeth than our dentists. And if the people could be taught to do everything possible in the care of these most useful organs how much the standard of your works would be raised.

The plan I have suggested has been tried in our schools and I know from personal observation that it has resulted in much good. I hope that in other schools the dentists and teachers will work together for the greater benefit of our pupils and that professional men in general will be led to see to a greater degree their responsibility concerning public welfare.

Through the influence of the medical profession the great burden of public health has been thrown largely onto the public schools.

In a very short time physical culture and medical examinations will be common in every school. As yet dental education has been considered but incidentally, but we are ready to give you a proper place. The needs of modern society and civilization necessitate the dental profession. Almost the only avenue for you to reach the public in a way to accomplish material good is through the public schools and the doors must be opened for your instruction.

DISCUSSION.

Dr. Ferguson: I am sure none of us can take any exception to the position taken by the essayist. This society has always taken a right attitude toward this subject. How can individual practitioners engage in this work without exciting public prejudice or professional jealousies is a question that has troubled many conscientious practitioners who would like to place their professional services at the command of our educational bodies. Possibly in larger towns it is better to have a concerted and unanimous action by the practitioners of the town, in small places where there is but one practitioner it is a very simple matter. Our school physiologies contain no proper hygienic instruction for the mouth and teeth. This must be remedied. If it can not be incorporated in the text, then we must supply it separately in tracts or some other suitable form. Something could be done by visiting teachers' conventions or institutes and talking to the teachers, and lastly the parents should be instructed as to the value and importance of enforcing cleanliness of the teeth and mouth.

Dr. Roe: I have had some experience in visiting the schools in my town and have tried to explain to the children why their teeth decay and what they can do to prevent it. I make an effort to impart my instruction in such a way as appeals most strongly to their sensibilities. For instance, I ask all present who snore when asleep to raise their hands, and thus I get their interest, then I tell them what snoring is and what influence it has on the welfare of the teeth.

Dr. Henderson, a physician: Formerly with my turnkey I very materially abreviated the functions of the dentist, but recently I have put away my turnkey and now send my victims to the dental surgeon. I have no doubt I and other physicians are responsible for many dental cripples, and for one I am glad to turn this business into more competent hands. I am pleased as a member of our school board to commend the work which Dr. Roe has done for our school children the past year. I estimate it as of incalculable value. I don't see why any dentist who feels qualified and has a desire to undertake this work should be deterred, because he thinks that some one may misconstrue his motives. There is always some jealous soul ready, if you raise your hand above your head, to try to raise his just a little higher. Such small matters ought not to stand in the way of such a humane work.

Dr. Essig: To my mind this work must of necessity be done, the people are ready for it and expect it, and we shall be censured if we do not push the agitation of it in all our dental societies.

Dr. Rix: Several years ago I did this work in the schools of Dowagiac and with good results to all concerned. The children became enthusiastic and their parents interested, and I was financially benefitted. My greatest joy was to see that as a result of that work many boys and girls grew up in my town with perfect sets of teeth. I had no competitors in business at that time and therefore excited no jealousies.

Dr. O. E. Lanphear: A dentist of my acquaintance has the privilege of supplying to his town paper articles on the care of teeth, and he thinks he is accomplishing some good in that way.

Dr. Warboys: It would seem necessary where this work is to be done by several dentists in the same community that they should have some understanding among themselves as to what should be taught so that there might not appear to be any conflict of instruction.

Dr. S. M. White: It seems to me that the schools, while affording opportunities for propagating this matter, are not the only place where effort should be made. Most children do not go to school until they have begun to erupt the permanent set of teeth. We must get at them before this, and the only way is to get at them through the mothers.

Dr. E. A. Honey: The practical details of management are not difficult. The school authorities must first be consulted. Then the dentist must arrange each to give instruction on some special topic, and to a certain grade of pupils or school.

Dr. C. A. Wise: In Kalamazoo we did this work in the schools. We even went so far as to examine the mouths of the pupils and where the need was found we advised the child as to what should be done.

Dr. Cook: Something should be done for the poor or those unable to pay for professional services. I have a case in mind, that of a servant girl who applied to me several years ago for cure of toothache. She had very bad teeth and was unable to pay for any treatment. I filled her teeth with cement without charge. She has since improved her financial condition and I am now filling her teeth with gold and she is paying for them. She is very grateful to me for saving her teeth for her.

Dr. Hoff: Some one has asked the question as to how soon mothers should take their children to the dentist. I would certainly think it should be long before they are sent to a public school, and probably careful and prudent parents look after these matters long before this period. But in our public schools are the children of parents who have never taken adequate care of their own teeth and feel no special anxiety about their children's teeth unless disease with pain forces them to take notice of their condition. It is very likely that as a result of such work in the public schools as has been suggested the parents may be reached, and through their children they may be taught the value and necessity for some kind of habitual care of the mouth. I know of two practitioners in this State who give up every Saturday afternoon to free work for such deserving poor children as the superintendent of the schools of the town may recommend to them. They are doing this purely as a benevolent work; but I have no doubt that in time it will prove financially profitable. So I think that work done now in the public schools will not only be of great benefit to the pupils of the schools but in time will bring its financial return to those who give the thought and time necessary to do the work properly.

